# Clinical, Community, and Caregiver Perspectives on Schizophrenia in Africa: A Systematic Review–Insights for Culturally Adapted Global Mental Health Strategies

**DOI:** 10.1192/bjo.2026.11261

**Published:** 2026-06-30

**Authors:** Oluwatimilehin Amao, Nicholas Aderinto, Adetola Babalola, Ayokunnumi Adegboyega, Temitomi Oyedele

**Affiliations:** 1 Birmingham and Solihull Mental Health NHS Foundation Trust, Birmingham, United Kingdom; 2 Ladoke Akintola University of Technology, Ogbomoso, Nigeria; 3 Kornberg School of Dentistry, Temple University, Philadelphia, USA; 4 Department of Medicine and Surgery, Bowen University, Iwo, Nigeria

## Abstract

**Aims:**

1. To synthesize fragmented evidence: The study seeks to bring together diverse research from community, hospital, and caregiver-focused studies to create a unified understanding of schizophrenia in Africa.

2. To guide clinical practice and service delivery: The study aims to provide evidence-based insights that help develop culturally appropriate healthcare and support services.

3. To evaluate research methodology and design: The review aims to examine how studies are conducted, focusing on study designs, diagnostic criteria (such as ICD–10 or DSM), and analytical approaches used across the continent.

4. To identify gaps in current literature: The study aims to highlight areas where evidence is lacking–specifically regarding interventional studies, caregiver burden, and long-term psychosocial outcomes.

5. To inform international policy and practice: The study aims to draw lessons for global mental health strategies, including care for African diaspora communities in high-income settings such as the UK.

**Methods:**

We conducted a systematic review of observational and interventional studies on schizophrenia in Africa. Searches covered PubMed, Embase, PsycINFO, African Journals Online, and Web of Science (terms: “schizophrenia” AND “Africa” with no language restrictions). All studies provided were included, and overlapping cohorts were explicitly flagged but retained. Data were extracted using standardized tables aligned with PRISMA and MMAT-compatible reporting. Extracted variables included study design, setting, population characteristics, diagnostic criteria, data collection, and analytical approach.

**Results:**

A total of 24 studies (1987–2025) from East, West, Southern, and North Africa were included. Study designs comprised longitudinal cohorts (n=5), cross-sectional surveys (n=14), mixed-methods studies (n=1), and randomized or dyadic trials (n=4). Diagnosticapproaches varied, most commonly CIDI/SCAN, ICD–10, or DSM criteria. Community-based cohorts were concentrated in Ethiopia, while hospital-based studies dominated West and Southern Africa. Evidence on caregiver burden and psychosocial interventions is emerging but remains limited in scale and duration. Interventional studies are few, with small sample sizes and heterogeneity in outcome measures.

**Conclusion:**

The African schizophrenia literature demonstrates robust observational foundations but limited interventional and caregiver-inclusive evidence. Heterogeneity in diagnostic and outcome measures constrains comparability. To improve clinical care, research should prioritize standardized assessments, scalable psychosocial interventions, and caregiver-inclusive approaches embedded in routine services. Future efforts should collaborate with African-led initiatives and integrate findings into WHO guidelines to enhance equitable schizophrenia care worldwide.

